# Assessment of Copper and Heavy Metals in Family-Run Vineyard Soils and Wines of Campania Region, South Italy

**DOI:** 10.3390/ijerph18168465

**Published:** 2021-08-11

**Authors:** Valentina Roviello, Ugo Caruso, Giovanni Dal Poggetto, Daniele Naviglio

**Affiliations:** 1 Department of Chemical, Materials and Industrial Production Engineering (DICMaPI), University of Naples Federico II, Piazzale V. Tecchio 80, 80125 Naples, Italy; 2 Department of Chemical Sciences, University of Naples Federico II, Via Cintia 21, 80126 Naples, Italy; ugo.caruso@unina.it (U.C.); daniele.naviglio@unina.it (D.N.); 3 Ecoricerche s.r.l, Via Principi Normanni 36, 80143 Capua, Italy; giogiodp@hotmail.it

**Keywords:** copper accumulation, copper toxicity, wine analysis, heavy metals

## Abstract

Copper-based phytosanitary treatments are widely employed in viticulture for combating the fungal diseases of European grape (*Vitis vinifera* L.). Herein we evaluated copper accumulation in the soil of a 50-year-old still productive vineyard in South Italy in comparison with samples taken from a ‘control’ area in which grapevines had never been cultivated, as well from an abandoned vineyard, now planted with cereals and forage crops, both close to the main area under investigation. Even though the heavy metal contents detected were not of concern for soils nor for wine, Cu accumulates in the soil in amounts significantly higher than the (grapevine free) control and remains at detectable concentrations also in abandoned vineyards where spraying activities had ceased about 20 years before this study. Despite the long Cu residence times in soil, the wine produced with grapes of the same vineyard showed Cu levels low enough to be safely used for human consumption, probably due to mechanisms of metal precipitation occurring during wine maturation, which are typically accompanied by sedimentation processes in artisanal production. However, this should not diminish the urgency of decreasing the copper usage as antifungal remedy in viticulture to prevent copper contamination of the agricultural soils.

## 1. Introduction

Vine growing and wine-making have been prominent in Europe, and particularly in Italy, since antiquity [1]. Viticulture requires numerous phytosanitary treatments for vine disease management during the pre-flowering stage through 2–4 weeks after the flowering period [2,3]. Among the phytosanitary interventions, those directed to control downy mildew (caused by *Plasmopara viticola*) are based on copper-containing products, with rameic sulphate + calcium hydroxide/copper oxychloride being one of the most common treatments [4,5,6]. In turn, these products can be used in combination with other phytosanitary drugs. Typically, Cu content in the most common copper-based products for agriculture ranges from 35 to 50% (*w*/*w*) with amounts as high as 3–5 kg Cu per hectare of vineyard [7]. The total number of phytosanitary treatments needed yearly is in strong correlation with factors such as weather conditions, latitude, and grapevine breed, and rarely is lower than four with the dispersion of about 8 kg Cu per hectare/year [8].

The fate of copper from fungicides includes about 90% deposited in the soil through both direct dispersion and wash-out from leaf surface or branches in decomposition. When present in neutral or slightly basic soil, Cu leads to different compounds all converging with time toward the highly insoluble CuO (having a solubility product Ksp = 10^−19.51^, 298 K [9]). This leads to copper accumulation in the soil [10,11] and when pH locally decreases, for example in the proximity of the plant roots as a consequence of organic matter decomposition, Cu dissolves and can be more easily absorbed by plants [12]. Family-run vineyards can remain productive also after one century or longer [13]. Hence, Cu concentration in soils of traditional vineyards, and to a lower extent also of younger industrial vineyards, can be particularly high, leading to environmental contamination.

In fact, amounts as high as 300 kg of Cu per hectare are typically released to a vineyard soil after one century [14]. However, this estimation is approximate because it considers as additive the amounts of copper used yearly, while one should consider that part of copper is eliminated in waters for example through percolation in loose soils or washout mainly from sloping sites [15].

Several analytical methods were applied to vineyard soil characterization [16] with particular attention paid to Cu contents [17,18], whose detection is particularly important also in wine analysis [19,20]. However, to the best of our knowledge there are no comparative heavy metal assessments taking into consideration contemporarily 1—vineyards subjected to Cu-based phytosanitary treatments; 2—adjacent control plots; and 3—adjacent former vineyards where grapevine cultivation was discontinued tens of years before the study.

**Figure 1:**
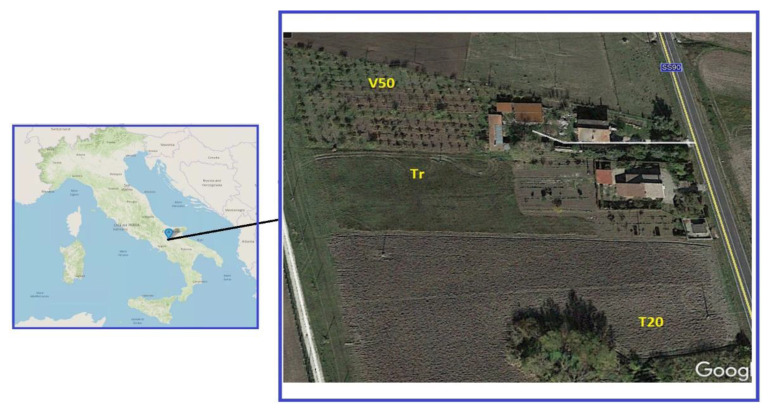
Map localization and satellite image with the area of study described in the present work. Images adapted from www.tuttocitta.it (**left**) (accessed on 10 August 2021) and Google Earth (**right**, Google, Maxar Technologies).

Hence, in the present study we report and compare data on the composition of a homogeneous soil from three adjacent sites within a radius of 100 m. These include a 50-year-old and still productive vineyard (indicated as V50, Figure 1); an adjacent field planted with cereals and forage since centuries (Tr); and a former vineyard (T20) adjacent to site 2, in production in the 1950–2000 period, now planted with rotating cereals and forage crops. V. Roviello, whose family inherited in part these plots, is aware of the reported information on the above-mentioned sites. In addition to the already mentioned soil analyses, we have extended our study also to grapevine branches and wines obtained from the site 1 (Figure 1).

## 2. Materials and Methods

### 2.1. Soil Sampling and Site Description

Samples used for the analyses were obtained mixing equal amounts (not less than 10 Kg each) of soil samples taken from ~20 cm deep from four equivalent points in the same plot at a mutual distance of about 50 m. In V50, which contains about 300 50-year-old vines, sampling was performed in the proximity of the grapevine trunk (sample 1A) and in the crossing point (1B) of the two diagonals of the square formed by four adjacent grapevines at a mutual distance of about 5 m. Similarly, for Tr and T20 we took the samples 2 and 3, respectively, obtained mixing four equivalent amounts of soil taken from four points that were equidistant from the vertices of the ideal rectangles enclosing the plots of land under exam. The physicochemical characterization of the soil in the area under investigation (territory of Ariano Irpino, Campania Region, Italy) was published recently [21] and shows that clay and silt are predominant accounting for more than 44% and 25%, respectively. On the other hand, pH is slightly basic (7.3), and N-total is about 0.9 g/kg [21]. Details including records from the local land registry (Cadaster) of the plots studied by us are reported in Table 1.

### 2.2. Grapevine Branches and Wine Samples

Grapevine branches (sample 4), the results of the annual pruning, were cut in January 2021, i.e., the same period in which we took the soil samples for this study. We obtained the A2000V (sample 7) sample from the 2000 vintage red wine (skin-fermented), and the following red wine samples, each from the 2020 vintage, with a volume of 2 × 1 L: A20V (sample 5): skin-fermented; A20SV (sample 6): fermented without any skin contact; C20: must from not sprayed grape fermented without any skin contact; A20V, A20SV and C20 were analyzed 6 months after the fermentation, following two settling steps on the naturally-clarified wine.

### 2.3. Chemical Analysis of Soil and Wine Samples

The chemical analysis of soil (1A, 1B, 2, 3, and 4) and wine samples (5, 6, and 7) was performed by inductively coupled plasma-optical emission spectrometry. Soil samples were dissolved in a HNO_3_ suprapure solution (Nitric acid 65% (*w/v*), Suprapure for trace analysis, Merck, VWR International Srl, Milan, Italy).

The metal content of all samples was investigated by microwave-assisted wet digestion (Microwave Mineralizer Speedwave4, Buchi, Flawil, Switzerland) according to EPA 3051A:2007 [22]. The determination was made using an Inductively coupled plasma-optical emission spectrometer (ICP-OES 5110, Agilent Tech, Santa Clara, CA, USA). The analyses of all samples were conducted based on calibration lines built according to EPA 6010 [23].

## 3. Results and Discussion

Grapevine cultivation requires, for disease control, frequent treatments with copper sulphate acting as a fungicide, especially in mixture with calcium oxide in the so-called “Bordeaux Mixture” [24,25], which is used for treatment of the vineyard object of the present analytical investigation. The soil under analysis was found to be clay-rich. The surface layer with a variable thickness of about 0.5 m is rich in humus and looks black in color: it is quite permeable to meteoric waters. The deepest layer, made up of yellow clay, absorbs water, and to a limited extent becomes impermeable.

Moreover, the site under study is flat and is not subject to any significant precipitation washout in the presence of abundant rainfall. Thus, both layers are effective absorbing and ion-trapping model systems.

### 3.1. Soil Analysis

**Figure 2:**
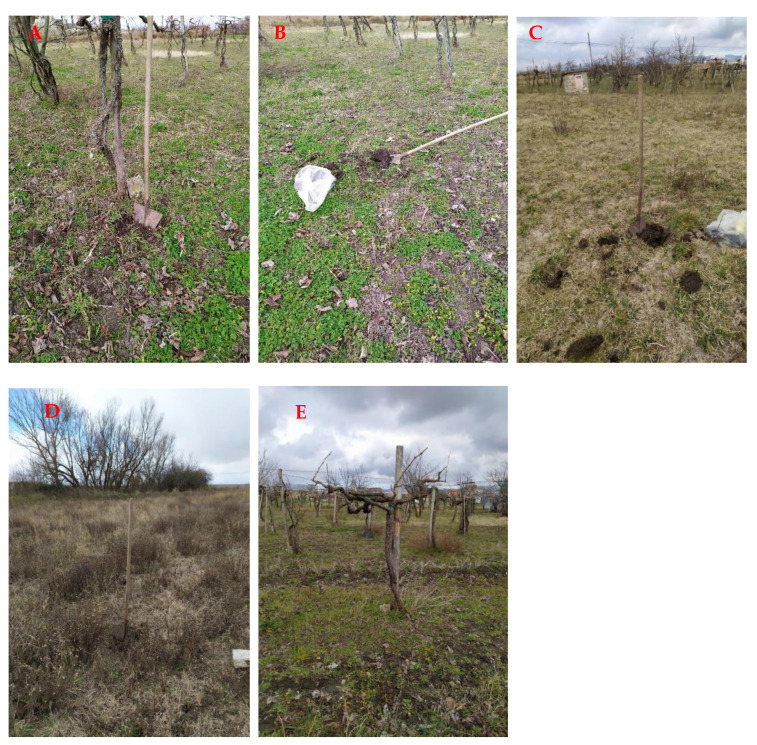
Sampling at the foot of the grapevines in V50 (**A**); in the middle of the grapevines in V50 (**B**); in a grapevine-free control area (Tr), (**C**); in ST20 (**D**). A 50-year-old grapevine specimen (**E**).

The chemical analysis (Table 2) of all soil samples taken as depicted in Figure 2 was performed by ICP-OES. Table 2 shows the results as ppm values of the various metals analyzed. The limit of quantification (LOQ) is 5 ppb, which means that below 5 ppb the readings are not reliable from a quantitative point of view, while they only give us information about the detectability. It can be observed that there is an abundant presence of Fe, Al, Mn, and Cu. On the other hand, Ca, Hg, K, Mg, Se, and Cr (VI) are present in quantities lower than the LOQ. The analysis results for the four samples with respect to all parameters but copper amounts are very similar and in line with the expected results [26]: We found high levels of aluminum due to the presence of aluminosilicates in clay, considerable amounts of iron, and also significant levels of manganese. Other ubiquitous elements were found to be present in moderate quantity (Co, total Cr, N, Pb, V, and Zn), whilst the most harmful were detected at low levels or below the detection threshold (As, Be, Cd, Hg, Tl, and Cr (VI)). As far as copper content is concerned, a significantly variable quantity for this metal was detected in our samples probably associated to anthropic activities.

Remarkably, higher levels of Cu were found in 1A soil sample, which we took at the foot of grapevines, as we expected as a consequence of the accumulation of this metal in the proximity of the trunk, deriving from the leaves fallen in autumn or by dripping of the copper-based fungicide during the phytosanitary treatments on the foliage. The soil sample (1B), taken by us as explained in Section 2.1, shows a Cu concentration that is about two thirds of that found in 1A, resulting from both fallen leaves and from mechanical activities of periodic soil tillage that lead to copper vertical and horizontal redistribution. However, these concentrations are four to six times higher than those detected in the reference soil sample (Tr), never subjected to any copper-based product treatment. Interestingly, we found a significant copper amount, four times higher than Tr, in the sample T20 taken from the site where grapevine cultivation was discontinued 20 years before the current study. This should be regarded as an average value for the soil of a former vineyard that underwent mechanical operations by plow or cutter for 20 years. The Cu concentration in T20 is still well-detectable probably because of a poor elimination capacity of the soil endowed with a scarce percolation and precipitation washout, and for the low Cu phytoextraction by spontaneous herbaceous plants or cultivated species (forage and cereals) [27]. Sample 4, constituted by branches obtained in late winter from the annual pruning (Figure 2E), did not show any significant levels of Cu, suggesting that the grapevine plants are able to absorb only limited copper quantities, and detected amounts are the result of the metal deposition on the vine bark after the phytosanitary treatments.

### 3.2. Wine Analysis

All wines samples were evaluated by ICP-OES analysis, without undergoing any treatment, leading to the detection of metals in the quantities indicated in Table 3, which shows the results as ppb values of the various metals analyzed. Also, in this case, LOQ is 5 ppb. In general, we observed higher levels of presence of Fe, Al, and Mn than Be, Ca, Hg, K, Mg, and Cr (VI), which are present in amounts lower than the LOQ.

Normally Cu levels in wines are never high and, however, are lower than the limit fixed from wine legislation of 1 mg/L [28], because the metal is partially eliminated in the lees during the fermentation process in the form of insoluble salts. However, significant Cu amounts are often present in industrial wines due to copper releases from the winery equipment or, more often, from direct wine treatments with copper-containing products added to improve the odor [28].

Our wine analysis showed comforting results in the quantification of various elements, and especially of Cu. The most significant and remarkable of these, in fact, is on copper levels that are very low. This is particularly important also in consideration of the fact that industrial wines are often endowed with Cu levels much higher (>100 ppb [29]), deriving from the direct must treatment with copper as reported in the literature. On the other side, Cu levels found by us are in line with (or lower than) other analytical reports on homemade wines [30].

Remarkably, the wine sample A20V, from must fermented on the grape’s skins, which one could expect to lead to increased copper levels having been in direct contact with the sprayed copper products, showed the lowest value of 5.76 ± 0.04 ppb after the clarification. This suggests that while skins could enrich wine with copper deposited on their surfaces, on the other hand they lead to increased levels of organic compounds like polyphenols or tannins that could effectively cause metal precipitation, and in particular copper levels’ decrease. The observed value was similar (5.76 ± 0.04 vs. 6.96 ± 0.29 ppb) to that found in a wine produced in the same year from grapes of a disease-resistant grape variety (Chambourcin, JS 26-205 [28], data not shown) that underwent no copper spray.

## 4. Conclusions

Extensive use of copper-based products and fungicides in grapevine cultivation leads to copper accumulation in vineyards higher than soils where this culture was never grown. The regular and yearly spraying of the “Bordeaux Mixture” or other copper-based phytosanitary products led to Cu levels higher than control even in soils where grapevine cultivation and consequent copper spraying was discontinued 20 years before the analysis. Even though our study revealed a very low copper content in homemade wine (much lower than that typically found in commercial wines due to copper releases from winery equipment or for the intentional addition of Cu-based products to remove wine bad odors), the soil of a former vineyard still shows copper levels significantly higher than control soil samples after 20 years of ceasing the grapevine cultivation. This led us to reflect on the importance of limiting copper-based products in grapevine cultivation in order to protect the environment [29], benefiting leaf arthropods [15] and aquatic life, which are particularly impacted by copper accumulation [30]. In our view, the Cu reduction in grapevine phytosanitary treatments can be achieved by using alternative fungicides based for example on plant extracts such as neem and orange oil, and/or by growing fungus-resistant grape varieties including the so-called PIWI varieties [31,32]. In this regard, recent efforts were made to produce quality wines from the PIWI grapes, but several countries allow the production of wine for commercial purposes only from varieties belonging to the botanical species *Vitis vinifera*, thus excluding resistant cultivars whose cultivation, in our opinion, should be reconsidered also in Europe, considering the environmental, health, and cost benefits of PIWI. All together these approaches could bring about quality wine production with a lower environmental impact and a greater respect for aquatic life [30].

## Figures and Tables

**Table 1:** Details and description of the plots studied within a flat area situated in the territory of the municipality of Ariano Irpino (Campania region, Italy).

ID Site	V50	Tr	T20
**Latitudo** **(°N)**	41°11′35.92″	41°11′34.68″	41°11′31.65″
**Longitudo** **(°E)**	15°08′07.66″	15°08′07.51″	15°08′10.08″
**Land Surface** **Areas (sq.m)**	4.4	3.33	800
**Details** **from Cadaster**	*Folio number* 13, *Parcel number* 248	*Folio number* 13, *Parcel number* 247	*Folio number* 13, *Parcel number* 246

**Table 2:** Analysis of soil samples: listed values (as ppm) are means of triplicate determination ± SD.

Metal	Sample 1A	Sample 1B	Sample 2	Sample 3	Sample 4
Al	32,879 ± 350	35,967 ± 375	41,273 ± 435	34,141 ± 330	36.03 ± 0.50
As	13.41 ± 0.18	11.94 ± 0.15	11.26 ± 0.16	14.60 ± 0.20	0.29 ± 0.04
Be	3.36 ± 0.08	3.42 ± 0.10	3.52 ± 0.10	2.58 ± 0.05	<5 ppb
Cd	0.57 ± 0.03	0.63 ± 0.04	0.54 ± 0.03	0.50 ± 0.03	<5 ppb
Co	24.14 ± 0.30	18.85 ± 0.25	18.22 ± 0.28	12.65 ± 0.18	0.10 ± 0.03
Cr tot	24.50 ± 0.22	20.99 ± 0.17	22.22 ± 0.20	19.85 ± 0.18	0.44 ± 0.02
Cu	180.47 ± 2.22	129.27 ± 1.15	35.41 ± 0.88	121.63 ± 1.75	16.59 ± 0.45
Fe	22,925 ± 250	24,321 ± 330	25,725 ± 458	22,250 ± 290	45.40 ± 0.66
Hg	<5 ppb	<5 ppb	<5 ppb	<5 ppb	<5 ppb
Mn	1901.7 ± 20.3	1400.3 ± 17.8	1295.5 ± 15.9	910.53 ± 12.6	32.27 ± 0.49
Ni	26.78 ± 0.34	19.76 ± 0.29	20.53 ± 0.31	18.94 ± 0.33	0.37 ± 0.01
Pb	89.89 ± 0.95	81.50 ± 0.88	92.82 ± 1.06	73.94 ± 0.99	7.90 ± 0.30
Se	<5 ppb	<5 ppb	<5 ppb	<5 ppb	<5 ppb
Tl	5.72 ± 0.17	<5 ppb	<5 ppb	<5 ppb	9.89 ± 0.24
V	64.16 ± 0.79	70.70 ± 0.89	72.49 ± 1.01	56.65 ± 0.63	0.64 ± 0.05
Zn	75.74 ± 0.95	80.56 ± 1.11	69.19 ± 0.82	68.38 ± 0.77	66.99 ± 0.69
Cr VI	<5 ppb	<5 ppb	<5 ppb	<5 ppb	<5 ppb
Sb	3.37 ± 0.09	2.56 ± 0.06	2.62 ± 0.07	1.49 ± 0.03	1.71 ± 0.04

**Table 3:** Analysis of wine samples: Listed metal contents are means of triplicate determinations (as ppb) ± SD.

Metal	Sample 5	Sample 6	Sample 7
Al	55.7 ± 0.8	104 ± 3	66.2 ± 0.9
As	12.1 ± 0.5	8.4 ± 0.3	7.2 ± 0.4
Be	<5 ppb	<5 ppb	<5 ppb
Cd	<5 ppb	<5 ppb	<5 ppb
Cr tot	15.3 ± 0.3	<5 ppb	<5 ppb
Cu	5.76 ± 0.04	17.8 ± 0.2	12.0 ± 0.1
Fe	705 ± 10	258 ± 7	1020 ± 30
Hg	<5 ppb	<5 ppb	<5 ppb
Mn	70.4 ± 0.6	50.7 ± 0.4	99.2 ± 0.9
Ni	37.7 ± 1.4	<5 ppb	<5 ppb
Pb	<5 ppb	<5 ppb	9.5 ± 0.3
Se	20.5 ± 0.3	16.9 ± 0.5	9.1 ± 0.4
Tl	<5 ppb	<5 ppb	<5 ppb
V	<5 ppb	<5 ppb	<5 ppb
Zn	21.6 ± 0.2	37.4 ± 0.5	211 ± 15
Cr (VI)	<5 ppb	<5 ppb	<5 ppb
Sb	<5 ppb	<5 ppb	<5 ppb
